# Supplementary material for: A Global Screen for Assembly State Changes of the Mitotic Proteome by SEC-SWATH-MS
Source: Cell Syst. 2020 Feb 26;10(2):133–155.e6. doi: 10.1016/j.cels.2020.01.001 (PMC7042714; doi:10.1016/j.cels.2020.01.001)

Q9HD20 | AT131_HUMAN | ATP13A1 ATP13A KIAA1825 CGI−152
Monomer MW [kDa]: 132.955 Monomer expected elution fraction: 38

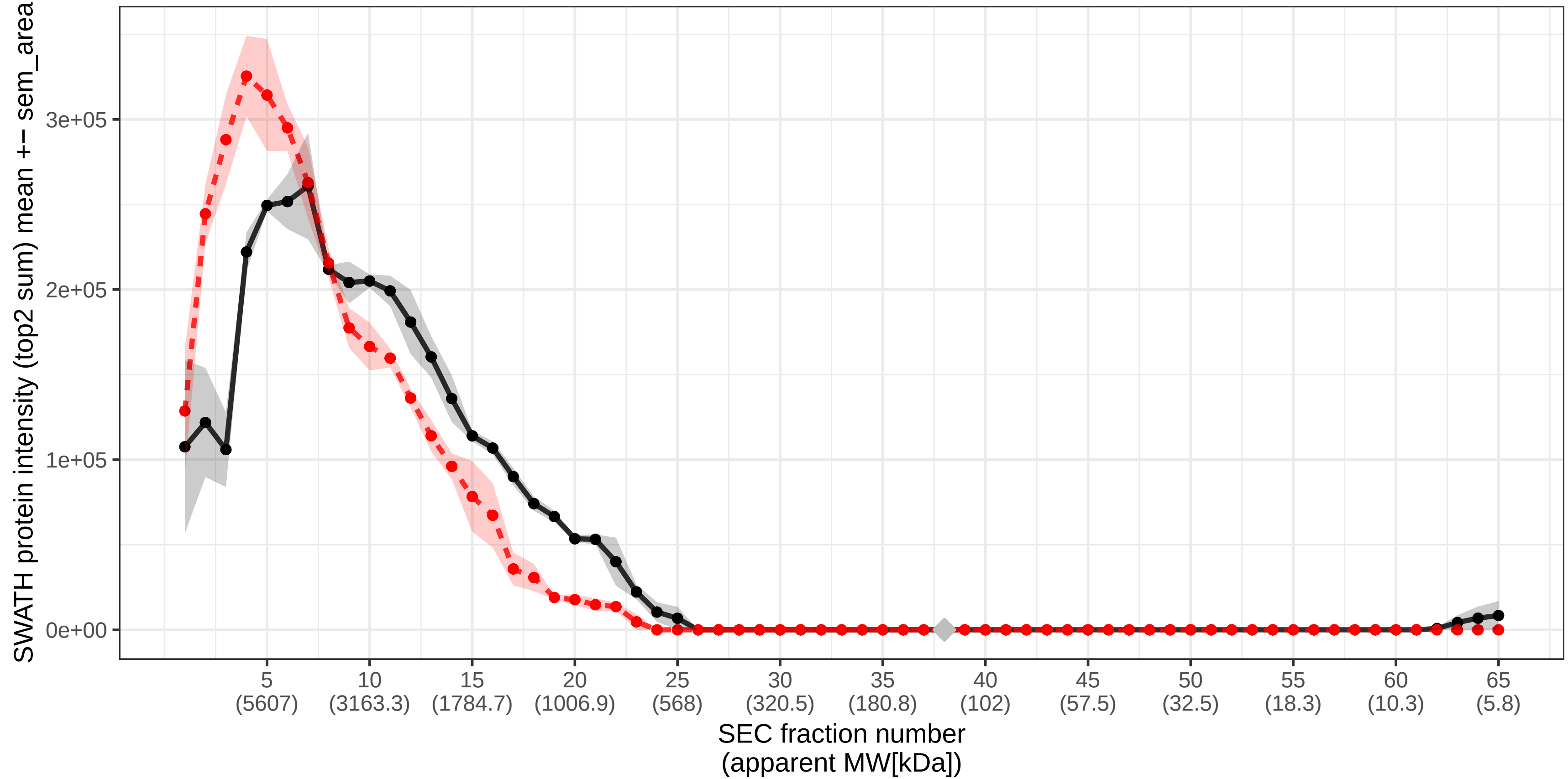

Supplement: Data S1. SEC-SWATH-MS Protein Chromatograms, Related to Figure 1 [file mmc6.zip › SECchrom_Q9HD20_AT131_HUMAN_ATP13A1_ATP13A_KIAA1825_CGI-152.pdf]
